# Radiomic analysis of contrast-enhanced CT for the prediction of microvascular invasion in hepatocellular carcinoma: literature analysis and practical challenges

**DOI:** 10.1093/bjro/tzaf032

**Published:** 2025-12-29

**Authors:** Sara Viganò, Pietro Andrea Bonaffini, Elisabetta De Bernardi, Andrea Corsi, Claudio Bandini, Eleonora Piccin, Clarissa Valle, Paolo Marra, Domenico Pinelli, Sandro Sironi

**Affiliations:** Department of Radiology, ASST Papa Giovanni XXIII Hospital, Bergamo, BG 24127, Italy; School of Medicine and Surgery, University of Milano-Bicocca, Milano, MI 20126, Italy; Department of Radiology, ASST Papa Giovanni XXIII Hospital, Bergamo, BG 24127, Italy; School of Medicine and Surgery, University of Milano-Bicocca, Milano, MI 20126, Italy; Medicine and Surgery Department, University of Milano-Bicocca, Monza, MB 20900, Italy; Interdepartmental Research Centre Bicocca Bioinformatics Biostatistics and Bioimaging Centre—B4, University of Milano-Bicocca, Vedano al Lambro,MB 20854, Italy; Department of Radiology, ASST Papa Giovanni XXIII Hospital, Bergamo, BG 24127, Italy; School of Medicine and Surgery, University of Milano-Bicocca, Milano, MI 20126, Italy; Department of Radiology, ASST Papa Giovanni XXIII Hospital, Bergamo, BG 24127, Italy; School of Medicine and Surgery, University of Milano-Bicocca, Milano, MI 20126, Italy; Department of Radiology, ASST Papa Giovanni XXIII Hospital, Bergamo, BG 24127, Italy; School of Medicine and Surgery, University of Milano-Bicocca, Milano, MI 20126, Italy; Department of Radiology, ASST Papa Giovanni XXIII Hospital, Bergamo, BG 24127, Italy; School of Medicine and Surgery, University of Milano-Bicocca, Milano, MI 20126, Italy; Department of Radiology, ASST Papa Giovanni XXIII Hospital, Bergamo, BG 24127, Italy; School of Medicine and Surgery, University of Milano-Bicocca, Milano, MI 20126, Italy; Department of Organ Failure and Transplantation, ASST Papa Giovanni XXIII, Bergamo, BG 24127, Italy; Department of Radiology, ASST Papa Giovanni XXIII Hospital, Bergamo, BG 24127, Italy; School of Medicine and Surgery, University of Milano-Bicocca, Milano, MI 20126, Italy

**Keywords:** hepatocellular carcinoma, radiomics, microvascular invasion, segmentation methods

## Abstract

**Background:**

Microvascular invasion (MVI) is considered an independent risk factor for early recurrence after curative resection of hepatocellular carcinoma (HCC). The ability to preoperatively predict MVI could lead to personalized treatment options in high-risk patients.

**Aims:**

To identify radiomic features from CE-CT that correlate with MVI in patients with HCC and evaluate the robustness and reproducibility of radiomic assessment by manual segmentation between readers with different experience.

**Methods:**

Clinical, CT imaging, and histological parameters were recorded. Sixty-two HCC lesions were manually contoured by three radiologists. Radiomic features were extracted. Features best correlating with angioinvasion were selected and assessed in univariate and multivariate models by means of 100 trials of 5-fold stratified cross-validation in terms of AUC, sensitivity, and specificity. The model identified on contours from the most experienced operator was then tested on contours from the other operators to assess inter-reader reproducibility.

**Results:**

Feature selection identified LI-RADS category and four arterial-phase radiomic texture features, with GLCM-ClusterShade and its high-frequency wavelet variant showing the highest predictive value for MVI. A bivariate logistic regression model combining these two features achieved an AUC of 79%, with 78% sensitivity and 64% specificity. The robustness of manual segmentation was strongly dependent on reader experience, and inter-operator reproducibility was suboptimal when the model was applied to contours from less experienced readers.

**Conclusion:**

Radiomics analysis may be able to predict MVI in patients with HCC. However, segmentation methods remain a practical challenge affecting reproducibility in radiomic studies.

**Advances in knowledge:**

This study, in agreement with the literature, identifies a radiomic model based on two textural features that could correlate with MVI in HCC. Furthermore, it aims to investigate some of the limitations in the application of radiomics in clinical practice, which still restrict it to a research setting, identifying an important limitation in manual segmentation methods. This aspect has not yet been sufficiently investigated in the literature.

## Introduction

Hepatocellular carcinoma (HCC) accounts for 75% of primary liver cancer and is the sixth most diagnosed cancer and the fourth leading cause of cancer-related death worldwide.^1^ Screening and surveillance programs for high-risk patients have led to early-stage HCC detection and curative treatment options.^2^ However, long-term survival remains unsatisfactory due to high recurrence rates, estimated being around 70% after 5 years from liver resection.^3^

One of the most important risk factors for early recurrence is the presence of microvascular invasion (MVI).^4^^,^^5^ Many studies have attempted to find either clinical or radiological parameters that could preoperatively predict MVI,^6^^,^^7^ leading to MVI prediction models combining “worrisome” imaging features both from CT and MR imaging and clinical-biological parameters.^8^ However, traditional imaging methods are limited by insufficient depth of imaging visual analysis and by subjective interpretation.

In the last decade, many studies have been concentrating on the role of radiomics, an emerging radiology field that aims to extract quantitative metrics (*radiomic features*) from radiologic images, based on the hypothesis that medical images reflect the underlying pathophysiological characteristics.^9^ It has been demonstrated that radiomics shows a high preoperative diagnostic performance for MVI status.^10^ Microvascular invasion prediction models based on radiomic features extracted from HCC segmentation from CT or MR imaging exams may thus provide an added value to clinical-radiological models. Nevertheless, radiomic studies need validation and standardization to be suitable for clinical practice.

Moreover, one of the primary practical challenges of radiomic studies is the lesion segmentation method, as most studies choose manual segmentation method, which must be tested for reproducibility and robustness between different readers. To date, only a few studies have investigated this aspect.^11^^,^^12^

On these bases, this study aims to investigate the contribution of radiomic analysis of contrast-enhanced CT (CE-CT) to the prediction of MVI in HCC patients who are candidates for curative surgical therapy in our centre. Additionally, the inter-reader reproducibility of manual segmentation of HCC lesions and the robustness of features extracted from CE-CT segmented by readers with different levels of experience in CT imaging were investigated.

## Methods

### Patients, treatment, and follow-up

This retrospective study included patients with suspected HCC at previous imaging who underwent an abdominal CE-CT before surgical treatment between March 2008 and November 2020. The inclusion criteria were as follows: (1) the presence of at least one HCC lesion, not previously treated with locoregional or systemic therapies; (2) available histopathological data from surgery that confirmed the presence of HCC; (3) available patients’ history and follow-up data; and (4) patients’ age older than 18 years old. Exclusion criteria were as follows: (1) poor image quality, unsuitable for accurate manual segmentation; (2) histological results other than HCC; and (3) the absence of angioinvasion data on the final histological report.

All patients underwent surgical treatment (lobectomy, segmental resection, atypical resection, liver transplant). Follow-up exams post-surgery, consisting of dynamic CT or MRI every three months for two years after surgery, and every six months later, were collected for all patients until December 15, 2021. Clinical data were also collected: (1) sex; (2) age at diagnosis; (3) presence of cirrhosis; (4) preoperatory plasma alpha-fetoprotein (AFP); (5) type of surgical intervention; (6) post-surgery relapse during follow-up; (7) age at relapse; (8) disease-free survival time (DFS); (9) date of last follow-up; and (x) age at last follow-up. The study was conducted in accordance with the Declaration of Helsinki and anonymity of patients was granted.

Among the 84 consecutive patients, 12 were excluded due to poor image quality, and 14 due to lack of data about angioinvasion on the final histological report. Therefore, the final study population included 58 patients and 62 HCC lesions (Table 1). Patients’ median age was 66.5 years (interquartile range, IQR 9.75 years). Among included patients, 27 (46.5%) had a cirrhotic liver at the time of HCC diagnosis. Concerning treatment, 32 patients underwent partial hepatectomy, 18 liver lobectomy, 7 atypical liver resection, and 1 liver transplant. Over a median follow-up time of 40.8 months (IQR 48.3 months), recurrence was observed in 36 patients (62%), with an average time of disease-free survival of 9 months (IQR 14.5 months).

**Table 1.** Main characteristics of the study population.

Population data	
Total, *n*	58
Age (years), median	66.5 (IQR 9.75)
Cirrhosis, total (%)	27 (46.5%)
AFP >400 ng/mL,^a^ total (%)	8 (4.3%)
Surgery, total (%)	
Partial hepatectomy	32 (55.2%)
Liver transplant	1 (1.7%)
Hepatic lobectomy	18 (18%)
Atypical liver resection	7 (12.1%)
Recurrence, *n* total (%)	36 (62%)

a Available for 54/58 patients.

Abbreviations: AFP = alpha-fetoprotein, measured in nanograms per millilitre (ng/mL); IQR = interquartile range.

### Histology

The description of the surgical specimens, aside from HCC confirmation, included: (1) predominant histological subtype (trabecular, trabecular with clear-cell aspects, trabecular pseudoglandular, mixed type), (2) tumour grade (G1, G2, G3), (3) the presence or absence of microvascular angioinvasion (MVI); and (4) the presence or absence of involved margins.

At histological analysis, 23 lesions (37%) were of grade G2, 15 lesions (24%) were G2-G3, and 19 lesions (30%) were G3. The grade was not specified in 5 lesions. The most frequent histological subtype was trabecular (28, 45%), followed by solid trabecular (11, 18%) and trabecular pseudoglandular (11, 18%), trabecular and clear-cell (7, 11%), and mixed type (5, 8%). MVI was present in 33 lesions (53%), and 13 lesions (21%) showed infiltrated margins (Table 2).

**Table 2.** Final histological data of resected HCC lesions (total 62).

Histological data	
Grade, *n* total (%)	
G1-G2	2 (3%)
G2	21 (34%)
G2-G3	15 (24%)
G3	19 (30 %)
Histological subtype, *n* total (%)	
Trabecular	28 (45%)
Solid trabecular	11 (18%)
Trabecular and clear-cell	7 (11%)
Trabecular pseudoglandular	11 (18%)
Mixed type	5 (8%)
Microvascular invasion, *n* total (%)	33 (53%)
Infiltrated margins, *n* total (%)	13 (21%)

### CT scan and qualitative evaluation

All patients underwent an abdominal CE-CT at a single institution, acquired on a 16-slice CT scan (GE LightSpeed VCT) from 2008 to 2012, and on a 64-slice CT scan (Philips Brilliance 64) from 2013 to 2021. A multi-phase liver CT protocol was applied, providing: (1) non-contrast phase of the upper abdomen; (2) late arterial phase (AP) of the upper abdomen with bolus tracking, with aortic triggering performed at L1, with a scan delay of 15-30 s after threshold aortic enhancement of 100-150 HU; (3) a portal venous phase (PVP) of the whole abdomen at 60-75 s after starting injection; and (4) delayed phase (DP) of the upper abdomen at 2-5 min after the start of the injection. Iodinated contrast medium (Iomeprol, 400 mgI/mL) was injected at a flow rate of 3-5 mL/s. Slice acquisition thickness was 1.5 mm.

Qualitative CT images evaluation was performed by one reader (V.S., third-year resident who completed CT rotation training) under the supervision of a senior staff member (P.A.B., with 10 years of experience in CT reading), including: (1) maximum tumour diameter on the axial plane (mm); (2) lesion density compared to normal liver parenchyma (isodense/hyperdense/hypodense) on different contrast phases; (3) the presence or absence of pseudo-capsule; (4) portal vein calibre (>13 mm was considered the cut-off for an indirect sign of portal hypertension); (5) portal vein thrombosis; (6) macroscopic signs of angioinvasion; (7) the presence or absence of satellite lesions; and (8) Liver Imaging Reporting and Data System (LI-RADS v2018) category.

Collected radiological qualitative data are listed in Table 3. The average maximum tumour diameter was 36.5 mm (IQR 38.75 mm). Imaging appearance on the different contrast phases was typical of HCC in most cases, with lesions isodense on the pre-contrast phase (34/62, 55%), hyperdense on the arterial phase (58/62, 93.5%), and with wash-out in portal venous (47/62, 76%) and late venous phases (79%, being this phase available for 34/62 patients). Concerning the lesions category, 45 (72.5%) were assigned LI-RADS 5 category, 9 (14.5%) LI-RADS 4, and 8 (13%) LI-RADS 3.

**Table 3.** Radiological qualitative data of HCC lesions (total 62) from CE-CT evaluation.

Radiological data	
Maximum tumour diameter, median (mm)	36.5 (IQR 38.75)
Imaging appearance	
PCP, *n*	25 hypo, 34 iso, 3 hyper
AP, *n*	0 hypo, 4 iso, 58 hyper
PVP, *n*	47 hypo, 8 iso, 7 hyper
DP,^a^ *n*	26 hypo, 6 iso, 1 hyper
Pseudo-capsule, *n* total (%)	28 (45%)
LI-RADS category, *n* total (%)	
LI-RADS 3	8 (13%)
LI-RADS 4	9 (14.5 %)
LI-RADS 5	45 (72.5 %)

a Available for 33/62 patients.

Abbreviations: IQR = interquartile range; AP = arterial phase; DP = delayed phase; PCP = pre-contrast phase; PVP = portal venous phase.

### Tumour segmentation and extraction of radiomic features

All lesions were independently manually contoured by three radiologists in training with different levels of experience in CE-CT imaging. *Operator 1* (V.S.) with two years of experience in evaluating HCC on CE-CT, *Operator 2* (P.E.) with one year of experience in evaluating CE-CT, and *Operator 3* (B.C.) with six months of experience in assessing HCC on CE-CT. The final segmentation results of *Operator 1* were validated by a senior radiologist with 10 years of experience (P.A.B.). The three radiologists in training manually contoured each lesion three-dimensionally (axial, coronal, and sagittal planes) using the segmentation software itk-SNAP on both arterial and portal venous phase. Before extracting the radiomic features, structures were smoothed, and voxels with HU <−100 were excluded. Radiomic features were extracted with Pyradiomics on arterial and portal venous phase CT images (FBS quantization with bin width 25 HU; FBN- 8 bin quantization on wavelet filtered images). Images were resampled on a common voxel grid of 0.8 × 0.8 × 0.8 mm^3^—for morphologic features and 2.0 × 2.0 × 2.0 mm^3^—for statistical and higher-order features. Overall, 490 features were extracted for each lesion.

### Statistical analysis

Multiple lesions in a patient were considered as independent. Radiomics features found to be significantly scanner-dependent according to the Kolmogorov-Smirnov 2-sample test were a priori excluded (55 features). The remaining 435 scanner-independent features were then considered in the statistical analysis. Additionally, the following selected clinical and radiological features were added: (1) age at diagnosis, (2) pre-operatory AFP serum level, (3) LI-RADS v2018 category, and (4) maximum tumour dimeter.

Lesions have been randomly divided 100 times into 5 equally sized subsets, ensuring a similar distribution of angioinvasion-positive cases in each. For each iteration, training groups were formed combining 4/5 subsets, resulting in 500 training sets. Feature selection was independently performed on each training set as described below. The association between features and angioinvasion was assessed using the non-parametric Mann-Whitney test. Features were ordered according to their *P*-values in ascending order. To reduce redundancy, inter-feature correlation was assessed using the Spearman correlation coefficient; features exhibiting an absolute Spearman correlation >0.5 with higher-ranked features were excluded. Subsequently, a LASSO logistic regression model (with L1 regularization and cross-validated penalty parameter) was applied for final feature selection within each set. To ensure the robustness and stability of the selected features across different data splits, only features selected in at least 30% of the 500 trials were retained. This frequency threshold was chosen to favour features consistently identified as relevant across multiple resampling, reducing the influence of variability in training subsets. The discriminative ability of selected features to identify lesions with angioinvasion was assessed with area under the curve (AUC); furthermore, by means of 100 trials of 5-fold stratified cross-validation, Youden thresholds were assessed in terms of sensitivity, specificity, positive predictive value (PPV), and negative predictive value (NPV).

As to multivariate analysis, all the possible combinations of 2-3 features from the selected feature pool were given in input to multivariate logistic regression models, which were assessed by means of 100 trials of 5-fold stratified cross-validation in terms of AUC, sensitivity, and specificity. The maximum number of features for multivariate models was defined according to the 10-events-per-variable rule.

It is worth noticing that feature selection, univariate analysis and multivariate analysis were performed on radiomics features computed on the contours provided by *Operator 1*, considered as reference. The best model identified on *Operator* 1 contours was then evaluated on features computed on *Operator 2* and *Operator 3* contours.

**Figure 1.**
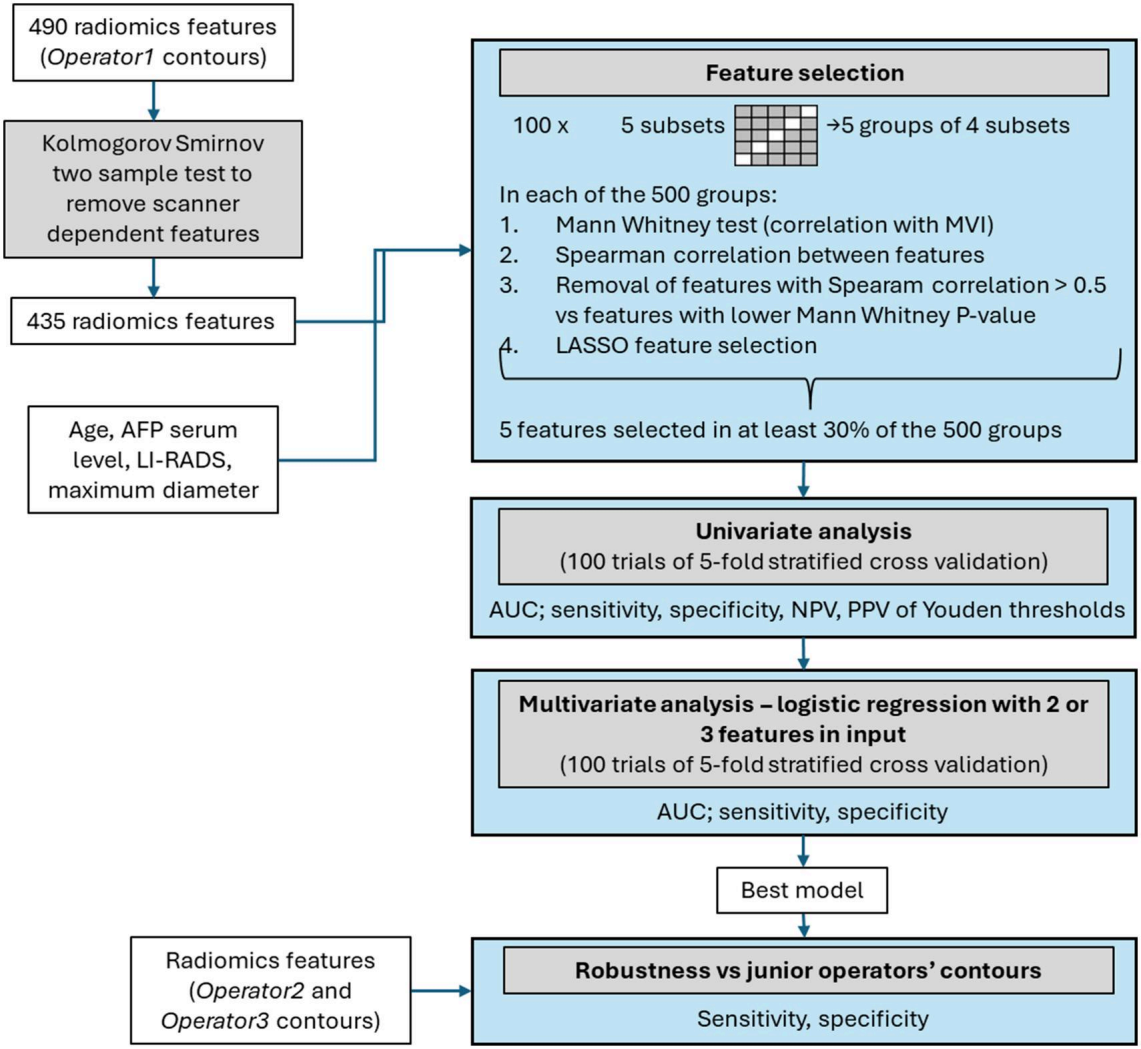
Statistical analysis diagram.

All the procedure is schematized in Figure 1.

## Results

### Feature selection

Features selected as the best correlating with MVI at histology were LI-RADS category (selected in 36% of the 500 subgroups) and 4 radiomic texture features computed on arterial phase images: *GLCM-ClusterShade*, *wavelet-HHH_GLCM-ClusterShade, wavelet-HHH_GLSZM-SmallAreaLowGreyLevelEmphasis*, and *wavelet-HHH_GLSZM-GreyLevelVariance*, respectively, selected in 85%, 77%, 41%, and 30% of the 500 subgroups.

### Univariate analysis

Univariate analysis results are presented in Table 4. The best AUC and sensitivity are associated with *GLCM-ClusterShade* (73% and 86%, respectively); however, the specificity is heavily suboptimal (14%). The same radiomic feature computed on the high-frequency wavelet band (*wavelet-HHH_GLCM-ClusterShade)* presents a better balance between sensitivity (63%) and specificity (62%), with a 68% AUC.

**Table 4.** Summary of univariate analysis derived from *Operator 1* segmentations.

Feature	*P*-value (whole sample)	AUC (mean—Std)	Sensitivity (mean—Std)	Specificity (mean—Std)	PPV (mean—Std)	NPV (mean—Std)	Youden threshold (mean)
*LI-RADS*	0.182	58%—3%	51%—43%	64%—33%	36%—31%	62%—22%	/
*GLCM-ClusterShade*	0.002	73%—3%	86%—25%	14%—30%	55%—12%	11%—21%	6.00
*wavelet-HHH_GLCM-ClusterShade*	0.014	68%—3%	63%—22%	62%—20%	66%—17%	62%—16%	0.005
*wavelet-HHH_GLSZM-SmallAreaLowGrayLevel Emphasis*	0.049	65%—3%	43%—23%	64%—28%	60%—22%	48%—16%	0.08
*wavelet-LLL_GLSZM-GrayLevelVariance*	0.029	66%—3%	43%—18%	85%—17%	79%—22%	57%—10%	3.21

Abbreviations: AUC = area under the curve; Std = standard deviation.

### Multivariate analysis

**Figure 2.**
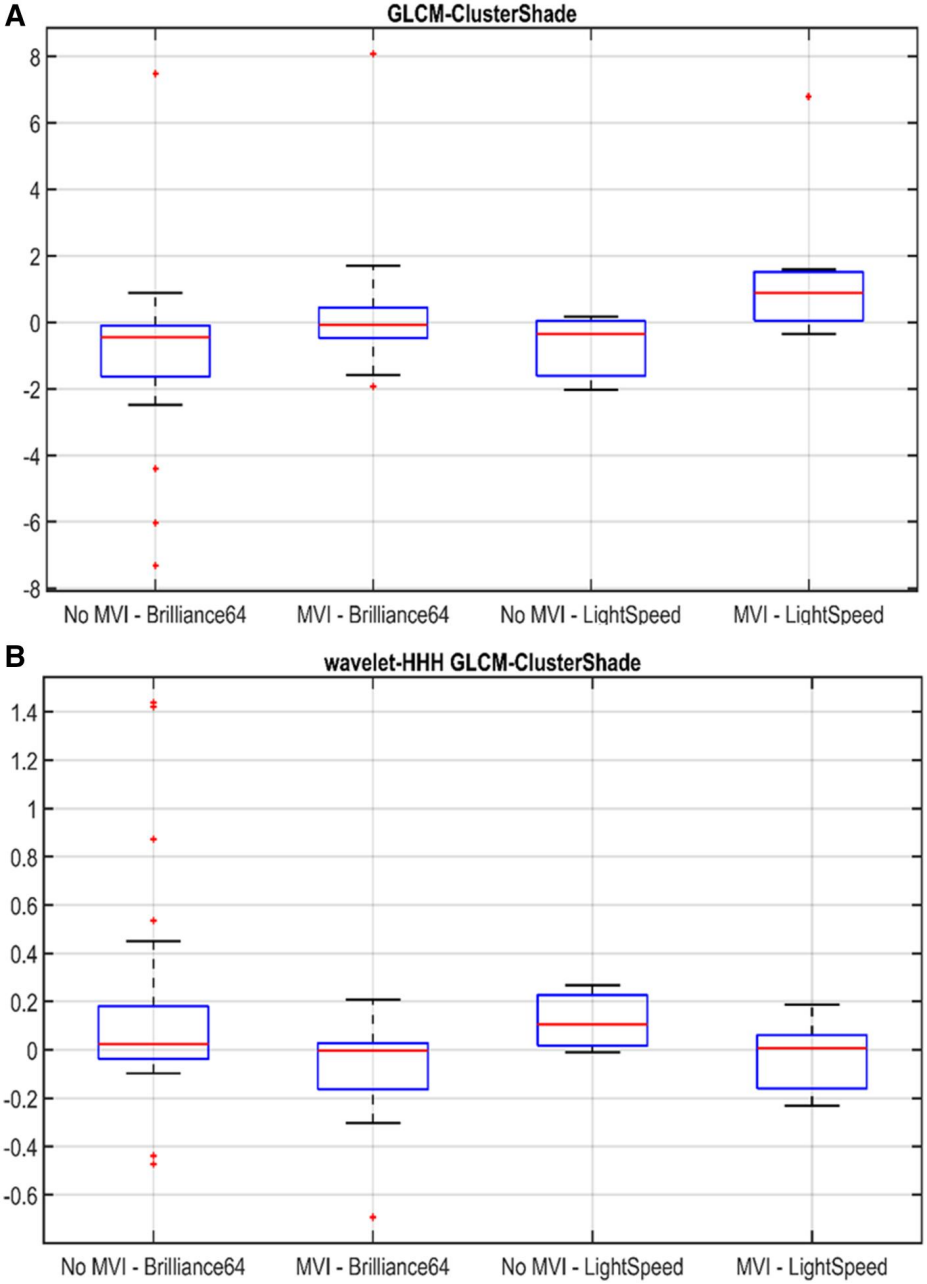
Boxplots for *GLCM-ClusterShade* computed on arterial phase original CT images (A) and on arterial phase CT images filtered on high-frequency wavelet band (B) from 2 different scanners, categorized by angioinvasion (MVI) and no angioinvasion (No MVI) groups.

**Figure 3.**
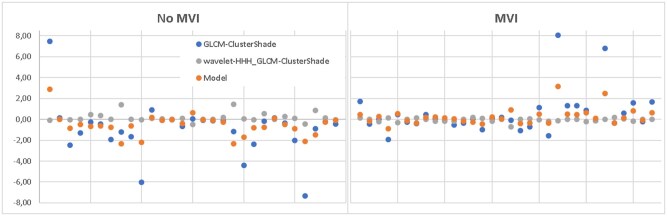
Multivariate best performing model. Features values and logistic regression model outputs are represented for no MVI lesions (left panel) and for MVI lesions (right panel).

A performance improvement (79% AUC, 78% sensitivity, 64% specificity) was obtained with a bivariate logistic regression model combining two of the selected arterial phase texture features, *GLCM-ClusterShade* computed on the original image, and on the high-frequency wavelet band (Table 5). In Figure 2, the distribution of the two features on lesions with and without MVI as a function of the CT scanners is represented. In Figure 3, the features values and the bivariate logistic regression model output are shown.

**Table 5.** Results from multivariate analysis derived from *Operator 1* segmentations.

Multivariate model—logistic regression	AUC (mean—Std)	Sensitivity (mean—Std)	Specificity (mean—Std)
0.37* *GLCM-ClusterShade—*1.32*wavelet-HHH_GLCM-ClusterShade	79%—12%	78%—15%	64%—18%

Abbreviations: AUC = area under the curve; Std = standard deviation.

### Inter-operator reproducibility

*Operator 2* and *Operator 3* structures were compared to *Operator 1* structures in terms of Dice score: 7/62 structures of *Operator2* and 27/62 structures of *Operator3* had a Dice score value <0.85.

Radiomic features were extracted from manual segmentations of HCC lesions provided by *Operator 2* and *Operator 3*. The best bivariate model identified on *Operator 1* data*,* considered the gold standard, was computed on those features. Results in terms of sensitivity and specificity on the whole dataset are reported in Table 6.

**Table 6.** Specificity and sensitivity of the bivariate logistic regression model identified on *Operator 1* data, if computed on features extracted in *Operator 2* and *Operator 3* contours.

Sensitivity test *Operator2*	Specificity test *Operator2*
81%	48%
Sensitivity test *Operator3*	Specificity test *Operator3*
91%	41%

## Discussion

Microvascular invasion is defined as vascular invasion by tumour cells, detectable only by microscopy,^13^ and observed in both portal and hepatic veins, possibly responsible for intrahepatic and distant metastatic spread. Microvascular invasion is considered an independent risk factor for early HCC recurrence within 2 years after curative tumour resection.^14^ Therefore, preoperative non-invasive prediction of MVI could be critical for developing personalized treatment strategies in patients with HCC, including neoadjuvant antiviral therapy in patients with active HBV infection and adjuvant TACE after curative resection for MVI-positive HCC.^5^

Many studies have been conducted to identify preoperative predictive factors of MVI among clinical-biological parameters and imaging characteristics from CE-CT and MRI with a liver-specific contrast agent analysis.

AFP is the biomarker employed in daily clinical practice for surveillance and early detection of HCC in high-risk patients. Recent studies have found an association between serum AFP levels >1000 ng/mL and the risk of MVI,^15^ even with a lack of specificity. Furthermore, in the context of liver transplantation (LT) due to HCC, serum AFP levels at the time of transplantation are considered strong predictors of HCC recurrence post-transplant. In this setting Magro et al identified a cut-off value of AFP >25.5 ng/mL at the time of LT, and an increase greater than 20.8% on the waitlist, as significant predictors of HCC recurrence following LT.^16^

Elevated PIVKA-II serum levels are also associated with HCC in high-risk patients. Some recent studies propose that it could be considered an independent risk factor for MVI, with a cut-off value >90 mAU/mL,^17^ with a non-completely known mechanism probably related to stimulation of neoangiogenesis.^18^

As for imaging and MVI correlation, Yang et al found that 2 LI-RADS imaging features (corona enhancement, mosaic architecture) and 2 non-LI-RADS imaging features (non-smooth tumour margin, peritumoural hypointensity on hepatobiliary phase) from gadoxetic acid-enhanced MRI could be used as preoperative biomarkers for predicting MVI in patients at high risk for HCC.^19^ Ranzulli et al identified 3 “worrisome” imaging features both from CE-CT and gadoxetic acid-enhanced MRI (non-smooth tumour margin, large tumour size, peritumoural enhancement) associated with a radiogenomic algorithm (based on the association between internal arteries and hypoattenuating halos at imaging and gene expression that they called 2-trait predictor of venous invasion, TTPVI) that could significantly predict the presence of MVI in HCC.^8^

Other promising results have been obtained by combining laboratory and imaging findings. Jiang et al recently proposed a preoperative MVI score integrating 5 imaging features from gadoxetic acid-enhanced MRI (non-smooth tumour margin, marked diffusion restriction, internal artery, hepatobiliary phase peritumoural hypointensity) and serum AFP >400 ng/mL.^20^

However, these studies show several limitations, as traditional imaging analysis is affected by insufficient depth of visual analysis by the human eye and by inter-reader variability due to subjective interpretation.

Since interest in the search of a possible preoperative predictor of MVI is steadily increasing, with a view to achieving personalized treatments in patients with HCC, many studies have been conducted to find a correlation between radiomic analysis and MVI.^10^ It has been found that the radiomic models correlating best with MVI at histology are based on textural features, and an improvement of performance is obtained when radiomic models are integrated with clinical-radiological models.

Ma et al developed a radiomic model mainly based on textural features from PVP CE-CT that exhibited good performance to predict MVI. Furthermore, they assessed that textural radiomic parameters could not be identified via visual inspection, being the radiomic model more objective than the PVP radiological characteristics assessed by radiologists.^21^ Bakr et al evaluated radiomic image features as a surrogate biomarker for MVI in HCC (AUC: 0.76, 95% CI 0.58-0.94), textural features being the most robust against inter-reader variability.^22^

Furthermore, Ma et al reported that a model incorporating the radiomic PVP model and the clinical model based on age, AFP, maximum tumour diameter, HBsAg, had a best predictive performance than the two models alone.^21^

Xu et al developed a CE-CT-based radiomic model integrating large-scale clinical, radiological factors, and radiomic features, mainly textural, to predict the MVI and outcomes in surgically resected patients with HCC with good performance (AUC: 0.909 in the training/validation set and 0.889 in the test set). Their study was based on a large cohort of patients. They considered in the clinical-radiological model the following parameters as predictor factors of MVI: (1) AST > 40 U/L, (2) AFP > 400 ng/mL, (3) non-smooth tumour margin, (4) extrahepatic growth pattern, (5) ill-defined pseudo-capsule, (6) peritumoural arterial enhancement, and (7) radiographic venous invasion.^23^

Liu et al proposed a nomogram model integrating LI-RADS features and quantitative radiomic signatures derived from CE-MRI to predict of MVI in HCC falling within the Milan criteria. The radiological score included baseline findings of HCC (tumour size >3 cm, non-smooth tumour margins) and LI-RADS ancillary features like mosaic architecture and corona enhancement. The radiomic score included mainly textural features. Moreover, the combined nomogram model exhibited improved performance in predicting MVI than the MRI-features model or the radiomic model alone.^24^

In agreement with the literature findings reporting a significant relationship between radiomic textural features and MVI, the present study shows that a radiomic model based on 2 textural features computed on the arterial phase of CE-CT images positively correlates with MVI at histology, with a 78% sensitivity and 64% specificity (assessed in cross-validation). The correlating textural features represent spatial relationships between neighbouring pixels: GLCM (grey-level co-occurrence matrix) is a matrix describing the frequency of pixels with the same grey-level pixel values in the image,^25^ which is assumed to objectively reflect intratumoural heterogeneity that could only be qualitatively assessed by radiological evaluation to a limited degree.^26^

In our experience, the LI-RADS category was the only radiological feature found to be an independent risk factor for MVI. Still, in our study no model integrating clinical, radiological, and radiomic features could robustly correlate with MVI, in contrast with the literature.

We do believe that the discordance between our results and literature findings in obtaining a combined clinical-radiological and radiomic model is mainly due to the small sample size of patients in our study. The limited sample size also importantly hindered our ability to evaluate the model using an independent dataset. The final sample size remained small even if conducted in a tertiary referral hospital centre, a reference for treatment and follow-up of high-risk patients with HCC and liver transplants. This was mainly due to the study’s retrospective nature, imposing us to include CE-CT images of HCC patients from our archives obtained by two scanners and with a non-standardized acquisition protocol. Due to the suboptimal quality of images, we excluded several lesions non-suitable for precise manual segmentation. Sample size and the retrospective nature thus represent critical challenges in radiomic monocentric studies, the majority of which is limited by a small sample size lacking uniform standards and external validation.^27^

Additionally, a known practical challenge of radiomics—investigated in this study—is HCC segmentation methods. Inter-operator reproducibility of manual 3D segmentation has been tested without satisfying results, particularly in terms of specificity (<50% in our results). We do believe that inter-operator non-reproducibility was mainly due to operators’ different experiences in CT imaging for HCC, being all radiologists in training with different timing of CT rotations. Comparing the structures of the 3 different operators, we found that the main differences between operators were for small lesions with a maximum diameter <2 cm. Another critical issue for manual segmentation was for lesions arising on cirrhotic liver parenchyma characterized by non-homogeneous enhancement, mainly as peritumoural transient hepatic attenuation differences (THAD), that could be difficult to interpret by less experienced operators.

We thus confirmed, according to the literature, that manual 3D segmentation, apart from being time-consuming, is non-robustly reproducible, therefore, unsuitable for application in clinical practice.

A few studies have been conducted to evaluate the best technique of lesion segmentation. Several efforts have been made to introduce a fully automatic segmentation system. To date, no automaticsegmentation system has obtained optimal results regarding reproducibility and thus reliability for clinical practice. Automatic liver segmentation systems are found to be inferior or comparable to experts’ manual segmentation. Furthermore, they are unsuitable for detecting and segmenting lesions with a maximum diameter ≤10 mm.^28^^,^^29^

Recent studies have been focusing on semi-automatic segmentation, which shows promising results. Qiu et al experimentally compared manual segmentation of HCC from CE-CT to two different semi-automatic segmentation methods to identify the differences in the reproducibility of radiomic features resulting from the impact of segmentation.^11^

Haniff et al investigated the reproducibility and robustness of radiomic features of HCC between manual and semi-automatic segmentation of HCC lesions from CE-MRI using 3D-slicer software. They found that, using the flood-filled algorithm, semi-automatic segmentation produced more reproducible and non-redundant radiomic features. They also analysed intra- and inter-operator reproducibility and found that experience of operators affects tumour segmentation, even when using semi-automatic segmentation. However, semi-automatic segmentation demonstrated high ICC values for intra- and inter-observers compared to manual segmentation.^12^

These are experimental studies, yet semi-automatic segmentation methods have the best potential in terms of reproducibility and robustness of extracted radiomic feature. Still, they are significantly affected by inter-operator variability based on the level of expertise, meaning that to obtain a reproducible and reliable segmentation system, segmentation should be performed by radiologists’ experts in hepatic imaging or trained in HCC segmentation.

In conclusion, our results showed that radiomic analysis has great potential in preoperatively predicting MVI in patients with HCC. Manual segmentation method resulted non-robustly reproducible and thus unsuitable for clinical practice. This study has several limitations due to its retrospective nature, small sample of patients, absence of an independent test set, and manual segmentation method. We find that these limitations represent a practical challenge affecting several radiomic studies. Thus, further multicentric prospective studies on a larger sample size and with a validated standardized segmentation system are warranted to obtain more robust clinical-radiological-radiomic models suitable for large-scale clinical practice.

## References

[1] Bray F , Ferlay J, Soerjomataram I, Siegel RL, Torre LA, Jemal A. Global cancer statistics 2018: GLOBOCAN estimates of incidence and mortality worldwide for 36 cancers in 185 countries. CA Cancer J Clin. 2018;68:394-424. 30207593 10.3322/caac.21492

[2] Harris PS , Hansen RM, Gray ME, Massoud OI, McGuire BM, Shoreibah MG. Hepatocellular carcinoma surveillance: an evidence-based approach. World J Gastroenterol. 2019;25:1550-1559. 30983815 10.3748/wjg.v25.i13.1550 PMC6452232

[3] Erridge S , Pucher PH, Markar SR, et al Meta-analysis of determinants of survival following treatment of recurrent hepatocellular carcinoma. Br J Surg. 2017;104:1433-1442. 28628947 10.1002/bjs.10597

[4] Sumie S , Kuromatsu R, Okuda K, et al Microvascular invasion in patients with hepatocellular carcinoma and its predictable clinicopathological factors. Ann Surg Oncol. 2008;15:1375-1382. 18324443 10.1245/s10434-008-9846-9

[5] Erstad DJ , Tanabe KK. Prognostic and therapeutic implications of microvascular invasion in hepatocellular carcinoma. Ann Surg Oncol. 2019;26:1474-1493. 30788629 10.1245/s10434-019-07227-9

[6] Reginelli A , Vacca G, Segreto T, et al Can microvascular invasion in hepatocellular carcinoma be predicted by diagnostic imaging? A critical review. Future Oncol. 2018;14:2985-2994. 30084651 10.2217/fon-2018-0175

[7] Hong SB , Choi SH, Kim SY, et al MRI features for predicting microvascular invasion of hepatocellular carcinoma: a systematic review and meta-analysis. Liver Cancer. 2021;10:94-106. 33981625 10.1159/000513704 PMC8077694

[8] Renzulli M , Brocchi S, Cucchetti A, et al Can current preoperative imaging be used to detect microvascular invasion of hepatocellular carcinoma? Radiology. 2016;279:432-442. 26653683 10.1148/radiol.2015150998

[9] Tomaszewski MR , Gillies RJ. The biological meaning of radiomic features. Radiology. 2021;298:505-516. 33399513 10.1148/radiol.2021202553 PMC7924519

[10] Li L , Wu C, Huang Y, Chen J, Ye D, Su Z. Radiomics for the preoperative evaluation of microvascular invasion in hepatocellular carcinoma: a meta-analysis. Front Oncol. 2022;12:831996. 35463303 10.3389/fonc.2022.831996 PMC9021380

[11] Qiu Q , Duan J, Duan Z, et al Reproducibility and non-redundancy of radiomic features extracted from arterial phase CT scans in hepatocellular carcinoma patients: impact of tumor segmentation variability. Quant Imaging Med Surg. 2019;9:453-464. 31032192 10.21037/qims.2019.03.02 PMC6462568

[12] Haniff NSM , Karim MKA, Osman NH, Saripan MI, Isa INC, Ibahim MJ. Stability and reproducibility of radiomic features based various segmentation technique on MR images of hepatocellular carcinoma (HCC). Diagnostics. 2021;11:1573. 34573915 10.3390/diagnostics11091573 PMC8468357

[13] Roayaie S , Blume IN, Thung SN, et al A system of classifying microvascular invasion to predict outcome after resection in patients with hepatocellular carcinoma. Gastroenterology. 2009;137:850-855. 19524573 10.1053/j.gastro.2009.06.003 PMC2739450

[14] Tabrizian P , Jibara G, Shrager B, Schwartz M, Roayaie S. Recurrence of hepatocellular cancer after resection: patterns, treatments, and prognosis. Ann Surg. 2015;261:947-955. 25010665 10.1097/SLA.0000000000000710

[15] Sakata J , Shirai Y, Wakai T, Kaneko K, Nagahashi M, Hatakeyama K. Preoperative predictors of vascular invasion in hepatocellular carcinoma. Eur J Surg Oncol. 2008;34:900-905. 18343084 10.1016/j.ejso.2008.01.031

[16] Magro B , Pinelli D, De Giorgio M, et al Pre-transplant alpha-fetoprotein >25.5 and its dynamic on waitlist are predictors of HCC recurrence after liver transplantation for patients meeting Milan criteria. Cancers (Basel). 2021;13:5976. 34885087 10.3390/cancers13235976 PMC8656660

[17] Poté N , Cauchy F, Albuquerque M, et al Performance of PIVKA-II for early hepatocellular carcinoma diagnosis and prediction of microvascular invasion. J Hepatol. 2015;62:848-854. 25450201 10.1016/j.jhep.2014.11.005

[18] Wang SB , Cheng YN, Cui SX, et al Des-γ-carboxy prothrombin stimulates human vascular endothelial cell growth and migration. Clin Exp Metastasis. 2009;26:469-477. 19263229 10.1007/s10585-009-9246-y

[19] Yang H , Han P, Huang M, et al The role of gadoxetic acid-enhanced MRI features for predicting microvascular invasion in patients with hepatocellular carcinoma. Abdom Radiol (NY). 2022;47:948-956. 34962593 10.1007/s00261-021-03392-2

[20] Jiang H , Wei J, Fu F, et al Predicting microvascular invasion in hepatocellular carcinoma: A dual-institution study on gadoxetate disodium-enhanced MRI. Liver Int. 2022;42:1158-1172. 35243749 10.1111/liv.15231 PMC9314889

[21] Ma X , Wei J, Gu D, et al Preoperative radiomics nomogram for microvascular invasion prediction in hepatocellular carcinoma using contrast-enhanced CT. Eur Radiol. 2019;29:3595-3605. 30770969 10.1007/s00330-018-5985-y

[22] Bakr S , Echegaray S, Shah R, Kamaya A, Louie J. Noninvasive radiomics signature based on quantitative analysis of computed tomography images as a surrogate for microvascular invasion in hepatocellular carcinoma: a pilot study. J Med Imag. 2017;4:1. 10.1117/1.JMI.4.4.041303 PMC5565686 28840174

[23] Xu X , Zhang HL, Liu QP, et al Radiomic analysis of contrast-enhanced CT predicts microvascular invasion and outcome in hepatocellular carcinoma. J Hepatol. 2019;70:1133-1144. 30876945 10.1016/j.jhep.2019.02.023

[24] Liu HF , Zhang YZZ, Wang Q, Zhu ZH, Xing W. A nomogram model integrating LI-RADS features and radiomics based on contrast-enhanced magnetic resonance imaging for predicting microvascular invasion in hepatocellular carcinoma falling the Milan criteria. Transl Oncol. 2023;27:101597. 36502701 10.1016/j.tranon.2022.101597 PMC9758568

[25] Park HJ , Park B, Lee SS. Radiomics and deep learning: Hepatic applications. Korean J Radiol. 2020;21:387-401. 32193887 10.3348/kjr.2019.0752 PMC7082656

[26] Liu Y , Liu S, Qu F, Li Q, Cheng R, Ye Z. Tumor heterogeneity assessed by texture analysis on contrast-enhanced CT in lung adenocarcinoma: association with pathologic grade. Oncotarget. 2017;8:53664-53674. 28881840 10.18632/oncotarget.15399 PMC5581139

[27] Yao S , Ye Z, Wei Y, Jiang HY, Song B. Radiomics in hepatocellular carcinoma: a state-of-the-art review. World J Gastrointest Oncol. 2021;13:1599-1615. 34853638 10.4251/wjgo.v13.i11.1599 PMC8603458

[28] Chlebus G , Schenk A, Moltz JH, van Ginneken B, Hahn HK, Meine H. Automatic liver tumor segmentation in CT with fully convolutional neural networks and object-based postprocessing. Sci Rep. 2018;8:15497. 30341319 10.1038/s41598-018-33860-7 PMC6195599

[29] Hänsch A , Chlebus G, Meine H, et al Improving automatic liver tumor segmentation in late-phase MRI using multi-model training and 3D convolutional neural networks. Sci Rep. 2022;12:12262. 35851322 10.1038/s41598-022-16388-9 PMC9293996

